# Can pleiomorphic psychotic symptoms with movement disorders mask wilson’s disease?

**DOI:** 10.1192/j.eurpsy.2022.1982

**Published:** 2022-09-01

**Authors:** J. Marques, M. Bajouco

**Affiliations:** 1 Centro Hospitalar Universitário do Algarve, Psychiatry, Portimão, Portugal; 2 Centro Hospitalar Universitário de Coimbra, Cri Psiquiatria, Coimbra, Portugal

**Keywords:** Wilson’s disease, PSYCHOTIC DISORDERS, First Episode Psychosis

## Abstract

**Introduction:**

Wilson’s disease is a rare (1:30,000) autosomal recessive disorder of copper metabolism that is caused by mutations in the adenosine triphosphatase copper transporting beta (ATP7B) gene, located on chromosome 13. The reported percentage of patients with psychiatric symptoms as the presenting clinical feature is 10%-20%.

**Objectives:**

To present and discuss a rare case admitted in the First Psychotic Episode Inpatient Unit (UIPEP) with pleiomorphic psychotic symptoms and low serum copper and ceruloplasmin and high 24h urine copper.

**Methods:**

The data was collected through patient and family interviews, as well as from his medical record. We searched Pubmed using MeSH terms: psychotic disorders AND Hepatolenticular Degeneration.

**Results:**

A twenty-two years old male, without known psychiatric history presented in the Emergency Department with a myriad of psychotic symptoms: motor stereotypes/mannerisms, paranoid delusions and auditory hallucinations. He was admitted in UIPEP, started low-dose antipsychotic medication with good response. As part of the implemented protocol, he did a battery of exams, including Brain CT-scan, EEG, ECG and blood and urine analysis, in which low serum copper and ceruloplasmin stood out, leveraging the suspicion of Wilson´s disease. Therefore, 24h urine copper was done, with 140 mcg/d (reference range < 40 mcg/d). Brain MRI was normal and no Kayser–Fleisher rings were seen by a consulting ophthalmologist.

**Conclusions:**

Without proper treatment, Wilson’s disease is a progressive and fatal disease. Therefore, it’s of upmost importance to recognize the clinical signs that raise suspicion of this disorder, especially recent onset in young adult of miscellaneous psychotic symptoms with movement disorders.

**Disclosure:**

No significant relationships.

